# Glucose as a Major Antioxidant: When, What for and Why It Fails?

**DOI:** 10.3390/antiox9020140

**Published:** 2020-02-05

**Authors:** Andriy Cherkas, Serhii Holota, Tamaz Mdzinarashvili, Rosita Gabbianelli, Neven Zarkovic

**Affiliations:** 1 Department of Internal Medicine # 1, Lviv National Medical University, 79010 Lviv, Ukraine; 2 Department of Pharmaceutical, Organic and Bioorganic Chemistry, Lviv National Medical University, 79010 Lviv, Ukraine; golota_serg@yahoo.com; 3 Department of Organic Chemistry and Pharmacy, Lesya Ukrainka Eastern European National University, 43025 Lutsk, Ukraine; 4 Institute of Medical and Applied Biophysics, I. Javakhishvili Tbilisi State University, 0128 Tbilisi, Georgia; tamaz.mdzinarashvili@tsu.ge; 5 Unit of Molecular Biology, School of Pharmacy, University of Camerino, 62032 Camerino, Italy; rosita.gabbianelli@unicam.it; 6 Laboratory for Oxidative Stress (LabOS), Institute “Rudjer Boskovic”, HR-10000 Zagreb, Croatia; zarkovic@irb.hr

**Keywords:** glucose, pentose phosphate pathway, NADPH, redox balance, glycogen, glycolysis, stress resistance, insulin resistance

## Abstract

A human organism depends on stable glucose blood levels in order to maintain its metabolic needs. Glucose is considered to be the most important energy source, and glycolysis is postulated as a backbone pathway. However, when the glucose supply is limited, ketone bodies and amino acids can be used to produce enough ATP. In contrast, for the functioning of the pentose phosphate pathway (PPP) glucose is essential and cannot be substituted by other metabolites. The PPP generates and maintains the levels of nicotinamide adenine dinucleotide phosphate (NADPH) needed for the reduction in oxidized glutathione and protein thiols, the synthesis of lipids and DNA as well as for xenobiotic detoxification, regulatory redox signaling and counteracting infections. The flux of glucose into a PPP—particularly under extreme oxidative and toxic challenges—is critical for survival, whereas the glycolytic pathway is primarily activated when glucose is abundant, and there is lack of NADP^+^ that is required for the activation of glucose-6 phosphate dehydrogenase. An important role of glycogen stores in resistance to oxidative challenges is discussed. Current evidences explain the disruptive metabolic effects and detrimental health consequences of chronic nutritional carbohydrate overload, and provide new insights into the positive metabolic effects of intermittent fasting, caloric restriction, exercise, and ketogenic diet through modulation of redox homeostasis.

## 1. Introduction

The glucose level in blood is one of the most important homeostatic parameters and is strictly regulated [1]. The complex interplay of signals from central and autonomic branches of the nervous system, and the impact of multiple hormones and cytokines, all support coordinated glucose flows within the body according to the actual needs and availability, in order to maintain its concentration in a narrow range [2]. Since severe alterations of glucose metabolism take place in many diseases—including diabetes, that affects hundreds of millions of patients worldwide—there is a wealth of information about health effects and biochemical changes due to high (over 10.0 mM) or low (under 3.5 mM) glucose levels. The pathways of glucose metabolism and its regulation such as glycolysis/glycogenolysis, pentose phosphate pathway (PPP), gluconeogenesis, polyol pathway, insulin signaling pathway and many others are very well studied and their physiology and pathophysiology are firmly established. It is well documented that hyperglycemia is associated with oxidative stress and that the severity of diabetes correlates with the levels of accumulation of lipid peroxidation products, oxidatively modified proteins and advanced glycation products; therefore, glucose itself is recognized by many prooxidant factor. Short-time higher than physiological glucose levels (more than 10.0 mM) cause certain degree of damage due to increased rate of non-enzymatic glycation of proteins but are usually not life-threatening if blood glucose does not exceed 20.0 mM and is associated with diabetic ketoacidosis due to insulin insufficiency. In contrast, low blood concentrations (2.5 mM and lower) can cause severe brain damage and potentially death within the periods of time as short as 5–6 h [3]. Brain and particularly neurons are the most sensitive to glucose deprivation, while other tissues and cells show a wide divergence in resistance to hypoglycemia [1] that is very much dependent on their function, peculiarities blood flow and capability to store glucose in the form of glycogen.

In the present review we would like to focus on the other aspects of glucose metabolism that are not sufficiently addressed in the literature, namely physiological aspects of involvement of glucose and its stores in the form of glycogen in regulation/maintenance of redox balance in cells and tissues. The role of glucose as a fundamental source of reducing equivalents to antioxidant intracellular machinery very often underestimated or ignored due to its reputation of primary source of energy. Understanding the involvement of glucose in redox processes will enable not only better explanation of its metabolic role, but also will open new possibilities to address poorly understood nature of insulin resistance, and metabolic changes in diabetes overall. Our interpretation is also consistent with the mounting epidemiological evidence and can explain deleterious health effects of excessive dietary consumption of carbohydrates and sedentary lifestyle.

## 2. Basic Overview of Glucose Metabolism and Its Role in Maintenance of Redox Balance

It is well-known that most of the glucose in human metabolism is utilized intracellularly in glycolytic pathway with further degradation of products in the tricarboxylic acid (TCA) cycle in order to produce NADH and ATP [4]. Glycolysis is effectively activated by insulin in conditions of glucose abundance, and a number of intermediates are also used for synthesis of needed amino and fatty acids as well as other important metabolites [2]. However, in conditions of limited glucose supply and/or excessive metabolic needs there are numerous alternative ways to generate enough NADH and ATP, for example by the oxidation of fatty and amino acids, and the utilization of ketone bodies. Flexibility and interchangeability of cellular energy supply provides sustainable and at the same time variable flow of metabolites that is capable to accumulate them when the nutrients are in abundance and consume them in a most effective way when there is their deficit. In periods of starvation or glucose deficit, the activation of catabolic programs is capable to maintain energy production in most of the organs [5]. Since neurons do not accumulate glycogen and total accumulation of glycogen in central nervous system being extraordinarily low is limited to astrocytes, neurons rely on glucose supply from the bloodstream [6]. Interestingly, in periods of starvation the brain can effectively use ketone bodies as a primary fuel accounting for more than 75% of its energetic needs, pointing out the possibility that glucose may be used for other purposes in this case [7]. This is further confirmed by the observation that glycolysis in neurons is actively downregulated by proteasomal degradation of 6-phosphofructo-2-kinase/fructose-2,6-bis- phosphatase-3, preventing the utilization of glucose for bioenergetics purposes. This mechanism, as suggested by authors, spares glucose in neurons for maintaining antioxidant status, especially in conditions of limited glucose supply [8].

The importance of other major pathway of glucose metabolism, which to certain degree is an alternative or parallel to upper glycolysis, a pentose phosphate pathway (PPP) is also long known. It is believed that its major function is the generation of reducing equivalents in the form of NADPH needed for de novo lipogenesis, synthesis of DNA and aromatic amino acid [9]. Indeed, proliferating cells use most of the NADPH for DNA and fatty acid synthesis [10]. The other major functions of NADPH are the reduction in oxidized thiols and glutathione, generation of superoxide anion and hydrogen peroxide during respiratory burst to fight infections and to provide redox signals to regulate cell functions. In addition, it is also needed for detoxification of xenobiotics [11]. A growing number of publications point out rerouting of glucose into a PPP as a major protective mechanism employed to counteract acute and severe oxidative stress [9,12,13]. According to the calculations, the full oxidation of one molecule of glucose in the PPP yields 12 molecules of NADPH reduced from NADP^+^ [14]. This aspect highlights the extraordinary efficiency and prompt responsiveness of this mechanism in balancing redox homeostasis in conditions of acute oxidative challenge. Indeed, activation of redox-sensitive transcription factors such as Nrf2 or FOXOs, in response to oxidative stress will result in induction of antioxidant enzymes within hours [15], while rerouting of glucose into the PPP to generate reducing power for antioxidant enzymes takes place almost immediately [14]. With the use of ^13^C flux analysis in neurons, it was recently shown that glucose metabolism through the PPP may be much more significant than was previously estimated [16]. Moreover, the authors demonstrated that about 73% of produced labeled pyruvate was exported from neurons as lactate [16]. This may indicate that neurons remove glucose that cannot be fully utilized in TCA away from the cells. In case of increased functional activity, oxidative stress or glucose deficit during starvation, most of the glucose flux may be redirected into the PPP.

The PPP is a major source of NADPH; however, it is not the only one. Substantial amounts of NADPH are generated in folate-dependent NADPH-producing pathway [10] as well as by cytosolic isocitrate dehydrogenase and malic enzyme [11]. However, these sources are often coupled with synthetic pathways; for example, isocitrate is in abundance when glycolysis is activated and contributes to fatty acid synthesis, therefore, it is difficult to expect their substantial contribution to the regeneration of NADPH in cases of oxidative stress. To a certain degree, the metabolism of amino acids can compensate for functional lack of glucose, and contribute to the maintenance of NADPH, but this seems to be the mechanism with limited power under extreme exposures. Noteworthily, recently it was shown that malic enzyme and 6-phosphogluconate dehydrogenase (6PGD) form a hetero-oligomer to promote the activity of 6PGD, independently on activity of malic enzyme [17]. It is likely that the other structural and functional interactions may exist in the cells in order to couple synergistic metabolic processes in response to oxidative stress. The activity of alternative pathways provides robustness of NADPH supply and, to some extent, compensates for the deficit of PPP flux in patients with glucose-6 phosphate dehydrogenase (G6PD) deficiency—one of the most common genetic diseases in humans [18]. Patients with G6PD deficiency generally have no symptoms and their lifespan is not affected by disease, but it was shown that in addition to increased hemolysis they are less resistant to some poisonings [18] and have higher risk of diabetes and metabolic syndrome [19]. Glucose-6 phosphate (G6P) is an exclusive substrate for G6PD, a rate limiting enzyme of the PPP that can be supplied from extracellular space in the form of glucose, then phosphorylated by hexokinase in human organisms. Alternatively, glucose-1 phosphate released from glycogen—if the latter is available in the cell—is converted by phosphoglucomutase to G6P. Some tissues—namely in the liver, kidneys or intestine [7], and to some extent the glial cells—can generate glucose via gluconeogenesis. Furthermore, tumor cells may reverse glycolysis in order to maintain their biosynthesis in glucose-free conditions [20]. Noteworthily, the expression of most of the enzymes in the PPP is controlled by Nrf2, a redox sensitive transcription factor involved in the upregulation of antioxidant and detoxifying genes, the degradation of damaged proteins and metabolic reprogramming during stress [21], pointing out the tight conjugation of redox balance maintenance and glucose metabolism. In other words, on the cellular and organism levels, responses to local or systemic oxidative stress are associated with increased glucose release/production by the liver and subsequently hyperglycemia, which may be physiological adaptive response in healthy subjects and may also take place as a chronic metabolic deterioration in diabetic patients.

## 3. Oxidative PPP Is Thermodynamically More Favorable Compared to Upper Glycolysis under Conditions of Limited Glucose Supply

**Figure 1:**
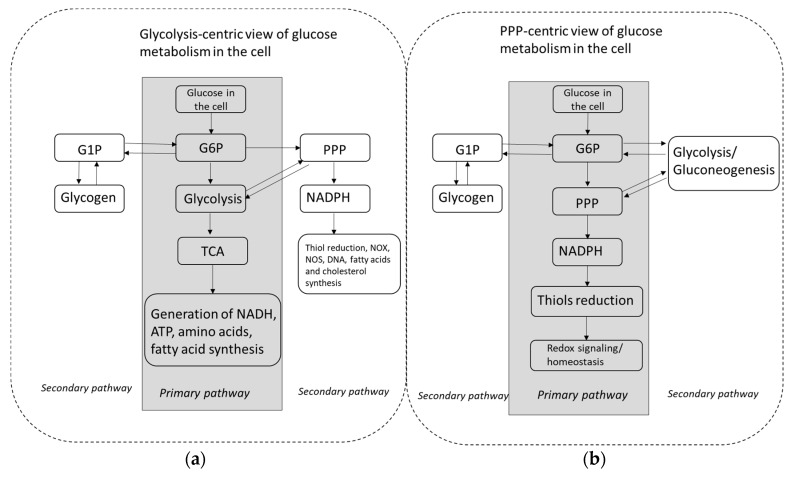
Schematic presentation of the conventional (**a**) and pentose phosphate pathway-centric (**b**) views of glucose metabolism. Abbreviations: G1P—glucose 1 phosphate, G6P—glucose 6 phosphate, PPP—pentose phosphate pathway, TCA—tricarboxylic acid cycle, NOX—NADPH oxidase, NOS—nitric oxide synthase.

In physiological conditions (without metabolic/oxidative stress) the ratio of reduced and oxidized forms of this coenzyme NADPH/NADP^+^ is very high (in the range of approximately 100/1, but is highly tissue-dependent) and the lack of free NADP^+^ prevents G6P from entering the PPP [14]. However, as soon as NADPH is oxidized (e.g., in conditions of oxidative stress) the increased availability of NADP^+^ immediately redirects metabolic flow to the PPP and suppresses further steps of glycolysis and the downstream utilization of glucose metabolites in TCA [8,13]. In other words, cells prioritize the metabolism of glucose through the PPP over standard reactions of upper glycolysis in order to maintain a sufficient NADPH/NADP^+^ ratio needed for the counteraction of acute oxidative challenge (prompt enzymatic reduction in glutathione and other oxidized thiols), biosynthesis and/or generation of superoxide during immune responses or as physiological redox signaling (Figure 1). Glucose availability for the PPP—either from extracellular space, or from intracellular glycogen stores—is essential for acute antioxidant responses ensuring the survival of cells and organism(s) in extreme conditions. Stable and robust liver glucose output in cases of severe stress (including oxidative stress), infection, starvation and extreme exercise is protected by the development of insulin resistance in order to provide sufficient glucose flow to balance redox homeostasis. In absolute quantities, especially at rest, glucose flow into the PPP may be low compared to standard upper glycolysis, but under conditions when the NADPH/NADP^+^ ratio drops, amounts of glucose entering the PPP will correspond to the degree of NADPH depletion.

**Figure 2:**
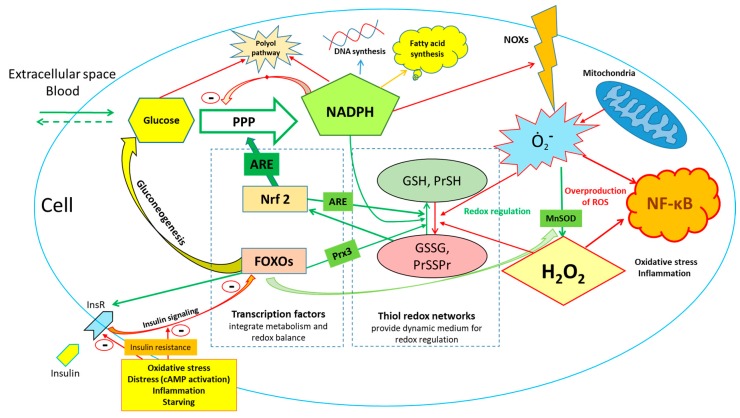
Glucose availability is a major factor in the maintenance of redox homeostasis through the reduction in oxidized NADP^+^, which is used for the subsequent reduction in oxidized glutathione and thiols. At the same time NADPH is used for synthesis of DNA and fatty acid synthesis and is needed for activities of NADPH oxidases, NO-synthase and other processes. The scheme is simplified and regulatory networks functioning in living systems are much more complex and include other mechanisms and feedback loops. For example, it was recently shown that the deletion of Nrf2 in mice can be—to a large extent—compensated by other adaptive mechanisms in conditions of caloric restriction [23]. This suggests that robust regulatory network beyond Nrf2 and FOXO transcription factors exists in order to maintain redox balance. Abbreviations: PPP—pentose phosphate pathway, NOX—NADPH oxidase, NOS—nitric oxide synthase, GSH—glutathione, GSSG—glutathione disulfide, Pr—protein, PrSSPr—disulfide bonds between/within the proteins and other molecules, ARE—antioxidant response element, InsR—insulin receptor, cAMP—cyclic adenosine monophosphate, FOXO—forheadkbox O transcription factors, Prx3—peroxiredoxin 3, Nrf2—Nuclear factor (erythroid-derived 2)-like 2 transcription factor, MnSOD—manganese superoxide dismutase, NF-kB—nuclear factor kappa-light-chain-enhancer of activated B cells.

A key factor leading G6P into the PPP is the presence of NADP^+^. The first reactions of the PPP as well as other reactions producing NADPH are energetically very favorable and are basically irreversible [22]. In contrast, most of the reactions of glycolysis are reversible. The activation of glucose utilization through glycolysis in physiological conditions takes place when the NADPH/NADP^+^ ratio is high (usually 50–100/1), glucose is relatively abundant and the insulin signaling is not compromised. Glycolysis serves as a source of pyruvate for TCA cycle and a number of anabolic intermediates in conditions of high carbohydrate availability, and is supposed to be fully activated only occasionally under physiological conditions, often switching to the oxidation of fatty acids when glucose availability is limited. This shift takes place in concert with activation of transcription factors involved in antioxidant defense (for example Nrf2 and isoforms of FOXO transcription factor) as a part of systemic antioxidant response aimed to balance redox homeostasis, where gluconeogenesis and activation of the PPP are fundamental parts of it (Figure 2). A sedentary lifestyle plus an abundance of carbohydrates in food lead to an imbalance of nutritional consumption and actual metabolic needs; therefore, redox dysregulation contributes to metabolic syndrome and diabetes type 2, and is an important factor that increases the incidence of cancer that is discussed in more detail in the next section.

## 4. The Epidemiological Evidence of Glucose Overload in Human Population: Current vs. Historical Nutritional/Behavioral Patterns and a Growing Potential of Pharmacological Interventions

As currently observed, massive nutritional carbohydrate overload associated with dramatic decreases in physical activity has caused an epidemic of non-communicable diseases such as obesity, metabolic syndrome, type 2 diabetes, atherosclerosis, hypertension and cancer [24]. A large-scale epidemiological cohort study pointed out that high carbohydrate consumption is a major factor for all-cause mortality, while different types of fat were not associated with increased mortality. Thus, authors question the current dietary guidelines suggesting an urgent need for their reconsideration [25]. On the other hand, the results of a large prospective cohort study indicate that health outcomes depend rather on quality of food rather than just limiting carbohydrate or saturated fat in the diet. In addition, “unhealthy” low carbohydrate and/or fat diet may increase all-cause mortality in studied population [26]. Together with other evidences, including numerous animal studies, a serious demand for strategies on how to counteract carbohydrate overload is indicated. Different approaches, including pharmacological interventions [27] as well as public health and food regulation policies, are increasingly discussed in the literature [28]. The careful evaluation of multiplicity of factors must be in place in order to determine the optimal nutritional pattern for health preservation and the prevention of age-related diseases.

In fact, an abundance of carbohydrates in food during human evolution was rather rare and more of a short-term and often seasonal luxury [29]. Human neonates grow very rapidly and their need for carbohydrate supply is probably higher than in adults than in other species due to the relatively large brain and rapid development of the nervous system; however, human breast milk “only” contains about 6.7 g per 100 mL of lactose accounting for up to 40% of calories—still the highest compared to other mammals [30,31]. It is not likely that adults need more carbohydrates than babies as a percent of calories intake in usual conditions without regular vigorous physical activity. The excessive consumption of carbohydrates and low physical activity are major contributors to increasing rates of metabolic syndrome and obesity in children and adolescents [32].

At high growth rate, the intensive physical activity as well as strong immunity needed for resistance to infections and occasional poisonings requires the maintenance of sufficient levels of glucose in blood, despite prolonged periods of carbohydrate deficit. Dietary glucose—as well as other carbohydrates—were precious food components with limited, often seasonal availability promoting survival under extreme conditions [29]. Therefore, an evolutionary sweet taste developed to detect sources of digestible carbohydrates [33]. Only the development of agriculture about 10,000 years ago enabled higher consumption of grain, increasing the share of carbohydrate in the diet. Even though carbohydrates became more available, any possible overload would rather not take place considering intensive physical activity of most of the people at that time. Thus, the gradual increase in basic food availability and elimination of physical work created a massive nutritional carbohydrate overload at the organism’s level causing respective health consequences [25]. Consistent with this, a metabolic core model was recently used to evaluate how increased glycolytic utilization of glucose together with glutamine-dependent lactate production promotes cancer growth [34]. Hyperglycemia may directly contribute to increased risk of cancer as it was recently shown by Wu et al. [35]. Overall, there is growing evidence—both mechanistic and epidemiological—that confirms previous predictions of interrelationships between risk of cardiovascular/metabolic diseases and cancer risks [36,37,38].

Consistent with this is recent data generated on a *C. elegans* model regarding the integration of stress-induced responses of nervous system with metabolic adaptations [39]. It was shown that the flight response mediated by tyramine (worm analog of catecholamines) in the end leads to the stimulation of insulin-IGF-1 signaling and has the opposite effect to longevity-promoting stress responses to heat, starvation, glucose restriction or exercise [40,41,42]. If hypothetically translated to modern humans, it can provide accurate mechanistical explanations as to why emotional stress accompanied with sedentary behaviors may have detrimental health consequences associated with both excessive insulin and adrenaline signaling causing atherosclerosis [24], contributing among the other factors to insulin resistance and accelerated aging [43], but can be reversed by exercise or fasting [44]. In this sense psycho-emotional stress prepares the organism to the impact of “expected” extreme factor in near future, and in cases of “false alarms”, the excess of glucose needs to be utilized by the activation of insulin signaling contributing to pathologic continuum that leads to atherosclerosis [24].

In regard to glucose balance/overload and its crucial role in metabolic diseases, new data obtained in clinical trials is very important, where patients were exposed to sodium glucose co-transporter 2 (SGLT-2) inhibitors. Designed initially to improve glycemia in patients with type 2 diabetes that are not optimally controlled by metformin monotherapy—canagliflozin, dapagliflozin, empagliflozin and others are continuously surprising clinicians and researchers by new positive effects far beyond glycemia, including—but not limited to—the improvement of insulin resistance, reducing body weight [45], preventing acute cardiovascular events [46], exerting a normalizing effect on blood pressure, kidney function [47] and reversing manifestations of heart failure [46]. A plethora of positive effects convincingly confirmed in strictly controlled clinical trials according to the highest standards of evidence-based medicine by simply getting rid of approximately 50–70 g of (excessive) glucose per day makes a significant difference for patients, and may potentially find its place among preventive interventions. It is worth pointing that some contribution to the effect may potentially come from sodium reabsorption inhibition, however, it is clear that the role of glucose excretion is prominent.

Thus, from the evolutionary point of view, the human organism was rather not used to the consumption of large amounts of glucose and has had to optimize its metabolism in order to be able to produce its sufficient amounts in accordance with metabolic needs. The importance of the PPP evolved in order to provide resistance to oxidative challenges that are crucial for survival in acute extreme conditions in multicellular organisms. The upregulation of PPP under oxidative stress is tightly coupled with enhanced glucose output from glycogen stores and/or stimulation of gluconeogenesis. Glucose 6-phosphate is a specific substrate for the PPP that makes glucose so important and strictly regulated in maintaining redox homeostasis in human organisms [1].

## 5. Glycogen Protects against (Not Only) Oxidative Stress

According to our hypothesis, availability of intracellular glycogen is supposed to be protective against oxidative stress, and vice-versa; its absence exposes cells to higher risk. Indeed, neurons, which are unable to accumulate glycogen appear to be among the most sensitive cells to oxidative stress, and they apply sophisticated mechanisms to direct the flow of glucose into the PPP in order to protect themselves [8]. Severe hypoglycemia may result in seizures, loss of consciousness, coma and—if glucose is not administered/ingested for longer periods (more than 5–6 h)—death [48]. Very similar clinical manifestations have been observed in cases of hyperbaric oxygen exposure (so called oxygen poisoning) that also causes severe redox imbalance in brain [49].

A recent *C. elegans* study demonstrated the crucial role of glycogen stores in resistance to acute oxidative stress [50]. Moreover, the excessive accumulation of glycogen from a high-glucose diet and with impaired glycogen degradation resulted in decreased lifespan of the worms [50]. Insecticide poisonings causing oxidative stress in the fruit-eating bat *Artibeus lituratus* causes glycogen stores depletion [51]. It was recently shown that hawkmoths, who have one of the highest metabolic rates among known animals, use nectar sugar directed through the PPP to counteract oxidative damage resulting from flight (extremely intensive exercise) [52]. In humans, the inability to deplete muscular glycogen in patients with glycogen phosphorylase deficiency (McArdle disease) is associated with severe exercise-induced oxidative stress and a risk of rhabdomyolysis [53]. This points out the possibility that the function of glycogen in muscles is not only as an energy store during periods of intensive contraction, but also for counteracting oxidative challenges associated with exercise.

It was noted that main life- and health-span promoting interventions such as caloric restriction, intermittent fasting and exercise have in common that the depletion of glycogen stores [44], thus reducing the protective capacity of glycogen and exposing the cells to moderate hormetic oxidative stress. Glycogen stores are not simply an intracellular source of glucose, they also have an important signaling function [54] and are protective against a number of stressful situations, namely hyper/hypo osmotic stress [55], anoxia/hypoxia [56]. In addition, growing evidence indicates that a metabolic switch from utilization of glucose, which is abundant in western diets, to ketone bodies use derived from fatty acids is an evolutionarily conserved trigger-point responsible for health effects from intermittent fasting, caloric restriction and exercise [57]. Furthermore, a complex interplay of hormones including insulin, glucagon, leptin, adiponectin and others regulate metabolic adjustments in conditions of food abundance and deficit to provide needed glucose levels and energy in the organism [58,59,60].

## 6. Epigenetics and Posttranslational Protein Modification Modulate Oxidative Stress Responses

Epigenetics regulates gene expression, modifying DNA methylation and chromatin structure. This regulatory mechanism works differently in each tissue to guarantee specific genetic responses to environmental factors (i.e., nutrition, chemicals, stress), without any changes in the sequence of nucleotides [61,62]. Epigenetics plays a key role starting from early life, where it is the master director of cell differentiation, X-inactivation and the programming of adult health [63]; epigenetic changes can be transferred to the progenies and, sometimes, they can be reverted [64].

DNA methylation consists of the methylation of Cytosine at CpG islands in the promoter region of genes which has been associated with gene silencing, while different responses (activation or inhibition of gene expression) derives from the methylation of CpG islands located in the regulatory regions of genes. Histone modifications are changes that are more complex, because functional groups (i.e., acetyl, methyl, P, etc.) deriving from oxidation of nutrients, can be added to histones’ amino acid residues, thus remodeling chromatin. The final result of histone modification is chromatin remodeling at specific genes, leading to increased/decreased gene expression associated with healthy or unhealthy regulatory responses [63].

Oxidative stress related with metabolic responses linked to hyperglycemia can enhance DNA methylation interfering with S-adenosyl-L-methionine (SAM), the key methyl donor for the DNA methyltransferases (DNMTs) which catalyze CpG methylation [65]. In particular, the deprotonation by superoxide anion of cytosine C5 at CpG islands can support the formation of DNA-SAM complex leading to the final cytosine methylation; furthermore, DNA methylation has been associated with the increase in DNMT1 and DNMT3B expression due to reactive oxygen species (ROS) [65,66]. Glucose can mediate epigenetic modification not only through ROS, but also because the high level of glucose can interfere with DNA demethylation via TET2 and AMPK [67].

However, oxidation at the level of guanine leading to 8-hydroxydeoxyguanosine (8-OHdG) in CpG islands, can also decrease cytosine methylation and reduce the binding of transcription factors to the promoter region. The oxidation of 5-methylcytosine (5mC) due to Ten-Eleven Translocation (TET) proteins leads to 5-hydroxymethylcytosine (5hmC) formation, which is deaminated to 5-hydroxymethyluracil and then replaced with unmethylated cytosine [68]. Oxidative stress can also inhibit the NAD^+^ dependent deacetylase SIRT1 that controls inflammatory responses, lipid storage, telomerase activity, mitochondrial respiration and ROS production [69,70]. In this context, a high-fat/glucose diet that decreases NAD^+^ content can negatively regulate Sirtuin activity.

The regulation of responses to oxidative stress is complex and includes many mechanisms [71] such as oxidative modifications of macromolecules by ROS [72], signaling through lipid peroxidation and their products [73] and involvement of different transcription factors (i.e., mentioned above FOXOs and Nrf2) [74,75]. Considering ubiquitous expression of these transcription factors as well as their crucial cellular functions it is very difficult to modulate them by pharmacological interventions [72,76]. Similarly, lipid peroxidation products play important physiological functions, for example in gastrointestinal tract [77]. Considering the significance of the regulatory functions of 4-hydroxynonenal and other lipid peroxidation products they also start attracting interest as a target for pharmacological interventions in major stress-associated disorders [78].

## 7. The Evidence from Glucose-6 Phosphate Dehydrogenase Deficiency

G6PD deficiency is one of the most common genetic human diseases and affects more than 400 million people worldwide [18], therefore, much can be learned from the published evidence. Since G6PD is a gateway to such an important metabolic pathway as the PPP, dramatic consequences to the patients could be expected. However, this is very often not the case. As was already mentioned in the introductory section, these patients have few or no symptoms, with generally positive prognoses and their expected lifespan is no different than that of the general population [18]. There are two basic explanations for this evidence: first, most of the patients have a moderate degree of G6PD deficiency and the PPP is still functioning at some level and, also, alternative pathways generate sufficient amounts of NADPH; second, humans with modern lifestyles are exposed to relatively low intensity stressors and there is simply no need for acute responses to stress. In contrast, severe G6PD deficiency does indeed have a detrimental effect on the immune system and causes higher susceptibility to infections [79]. In addition, G6PDH-deficient athletes and patients with this genetic defect may have severe hemolytic crises after physical exertion [80]; however, the severity of susceptibility of individual subjects may vary widely [81]. Complete G6PD knockout in mammals is incompatible with life, but in embryonic mice stem cells it led to the severe susceptibility of cells to oxidative stress induced by hydrogen peroxide or diamide and reduced cloning efficiency. However, the later was restored when the oxygen concentration was reduced [82]. Therefore, in conditions of substantial oxidative challenge, the proper function of the PPP and generation of NADPH are essential for survival [83]. Conversely, G6PD overexpression may be expected to increase resistance to oxidative stress. Indeed, recent reports indicate that G6PD overexpression extends the lifespan of *Drosophila melanogaster* [84], which is consistent with some of the results obtained using a G6PD overexpressing mouse model, where it leads to the extension of the health-span of mice and increased resistance to oxidative damage [85].

## 8. Redox Dependence of Pancreatic Regulation of Blood Glucose Levels

A growing body of evidence indicates that the release of insulin from pancreatic β-cells depends on the function of the PPP. It was shown that insulin levels in G6PD deficient patients are lower compared to unaffected controls, and these patients have significantly reduced insulin responses to the elevation of blood glucose [86]. More recently, with the use of metabolomics approach it was shown that insulin release is controlled by the direct implication of the PPP [87]. According to a recent review, among the most important amplifiers/regulators of insulin secretion by β-cells are high levels of NADPH and glutathione [88]. So, insulin release is taking place in conditions of “metabolic welfare” and oxidative stress may reduce the ability of β-cells to release insulin [89]. α-сells also have their intrinsic mechanisms of glucose sensing relying on intracellular redox balance, but they are activated by pro-oxidant situations [90]. Interestingly, under physiological conditions β-cells do not accumulate glycogen but are able to do so under prolonged hyperglycemia and may prolong insulin secretion even after normalization of glucose concentration. In contrast, α-cells do not accumulate glycogen, so when the concentration of blood glucose drops, they can be quickly activated without delay [91], which is extremely important in case of emergencies such as hypoglycemia.

One may argue that there are many other mechanisms for the regulation of insulin and glucagon that can either enhance or inhibit respective secretion, including paracrine δ-cells secreting somatostatin [92,93], effects of glucagon-like peptide-1 (GLP-1) [94], glucose-dependent insulinotropic peptide (GIP) [95], leptin/adiponectin axis [96], and autonomic nervous system [97,98]. However, the effects of all these regulators are integrated at the level of α- and β-cells and their metabolism resulting shifts of redox potential [87,88,90].

## 9. Inflammation, Insulin Resistance and Redox Homeostasis

NADPH produced by the PPP or by other pathways is also used by NADPH oxidases and nitric oxide synthase to produce superoxide anion. This is important for proper functioning of the immune system and for redox regulation of multiple processes in the tissues including endothelial function [99]. It was shown, that pro-inflammatory interleukin 1β enhances glucose uptake under hyperglycemic conditions in cultured human aortic smooth muscle cells. It also activates PPP and promotes the production of superoxide by NADPH oxidase contributing to vascular damage [100] pointing out a particularly dangerous combination of inflammation and hyperglycemia. Since immune cells require glucose for their function, they send regulatory signals, for example TNF-α [101] or microRNAs [102] to the liver to enhance hepatic glucose output and thus, chronic inflammatory conditions may cause insulin resistance [103]. The opposite effects are mediated by anti-inflammatory interleukin-10 [104] and the spleen plays a particularly important role in these regulatory interactions [105]. The autonomic nervous system may also be involved in regulation of interactions of local inflammatory conditions and oxidative stress [106]. Autonomic output is actively involved in the regulation of glucose homeostasis and can adjust the rates of glucose production and utilization independently of hormonal influences [107]. The healing of oxidative stress-associated conditions, therefore, may improve autonomic balance [108]. Chronic carbohydrate overload and reduced physical activity cause obesity and metabolic syndrome, and for these conditions, insulin resistance is very typical [109]. Thus, there exist complex multilevel regulatory interactions to provide a sufficient flow of glucose to tissues in order to maintain a redox balance. Prompt adjustments of redox homeostasis are critical for the immune defense where the PPP plays a major role. Insulin resistance developing in this case seems to be adaptive and to some extent a protective mechanism [110], but, if dysregulated, it leads to detrimental health consequences.

## 10. Glucose—“Oxidant” or “Antioxidant” after All? *Sola Dosis Facit Venenum*

There is a certain degree of confusion in the literature concerning the role of glucose in the maintenance of redox homeostasis. It was long known, that diabetes and hyperglycemia obviously cause redox dysregulation, oxidative stress and accelerated ageing [111]. On the other hand, glycogen clearly protects against the oxidative stress and at the same time decreases lifespan and health span [50,112]. Starvation and stress-induced gluconeogenesis clearly support survival and improve health and lifespans [112,113], but are associated with the increased generation of ROS [40]. Moreover, the complete withdrawal of glucose hence leads to short-term fall in ATP production, and surprisingly causes a rise in ATP and increased mitochondrial content shortly after that is associated with the activation of the protein deacetylase SIRT1. At the same time increased ROS production or possibly insufficient ROS utilization is documented [114]. This suggests that there are a variety of effective metabolic adaptations to compensate lack of glucose for ATP production, but a subsequent deficit of reducing power to counteract to increase in ROS production is much more difficult to compensate. Exercise, intermittent fasting/caloric restriction all lead to functional glucose/glycogen depletion through activation of autophagy and support healthy aging that requires certain degree of oxidative stress that leads to metabolic shift to catabolism and activate endogenous protective mechanisms that include enhanced protein quality control, stimulation of endogenous antioxidant defense including gluconeogenesis [57,111,115].

The other interesting aspect of glucose involvement in redox homeostasis is that supraphysiological concentrations of glucose in the cells may actually lead to the increased production of hydrogen peroxide by mitochondria through the inhibition of mitochondria-bound hexokinase [116]. Similarly, chronically high levels of NADPH may contribute to the enhanced generation of superoxide and/or hydrogen peroxide by NADPH-oxidases (NOX) as well as enhance the reduction in glucose in polyol pathway (Figure 2) [99]. Involvement of NOX in regulation and maintenance of redox homeostasis is very complex and depends on its isoforms that are expressed differently in tissues. For example, cardiomyocytes express NOX2 and NOX4 isoforms and use the generation of hydrogen peroxide for the regulation of cellular metabolism and contractile function (NOX4 activation acts similarly as beta-blockers decreasing inotropy) [117]. NOX isoforms are increasingly studied as potential therapeutic targets in order to modulate redox balance in the cells during cardiovascular and metabolic diseases [118].

Diabetes, both type 1 and 2 are well documented diseases associated with oxidative stress. The fundamental feature of diabetes is chronic hyperglycemia due to excessive glucose production and/or impaired its utilization by the tissues. There are several mechanisms contributing to oxidative stress in conditions of hyperglycemia that include but are not limited to non-enzymatic glycation and formation of advanced glycation products, the activation of polyol pathway that results in a depletion in NADPH (decreased rate of enzymatic reduction in oxidized glutathione and thiol groups of proteins), an increase in NADH and a depletion in NAD^+^ (increased superoxide anion production in mitochondria), the inhibition of histone deacylation and excessive histone acetylation due to the accumulation of acetyl-CoA, as well as the generation of excessive amounts of sorbitol and fructose [119].

**Figure 3:**
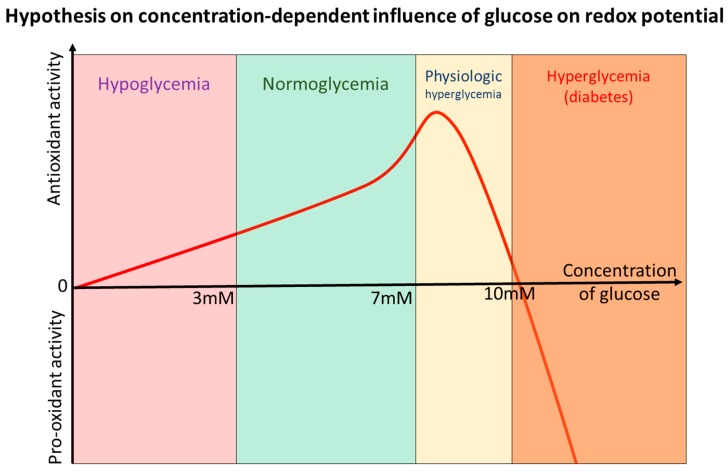
The dependence of redox effects of glucose on its concentration (hypothetic relationship suggested by the authors). Hypothetical simplified model describing the influence of the blood concentration of glucose on redox potential in human organisms. Multiple additional factors influencing redox potential such as concentration of oxygen, availability of amino and fatty acids, type of cells and effects of either hormones (insulin, glucagon) or cytokines are not considered. Concentrations of glucose as well as the shape of the curve are roughly estimated and not confirmed by actual experiments and may significantly vary depending on conditions and tissue type.

Taken together, the evidence indicates that there exists a delicate balance between the protective and damaging redox effects of glucose and chronic dietary carbohydrate overload may affect the regulatory mechanisms that developed during evolution to maintain redox homeostasis (Figure 3). Bell-shaped antioxidant activity of glucose explains well necessity to regulate it strictly within the narrow range, while both excessively high and/or low levels of glucose/carbohydrates lead to oxidative and metabolic stress that quickly becomes damaging [120]. The dysregulation of glucose metabolism that takes place in diabetes closes vicious cycle further exacerbating redox dysregulation that was actually meant to be fixed by induction of hyperglycemia.

## 11. Important Implications

**Figure 4:**
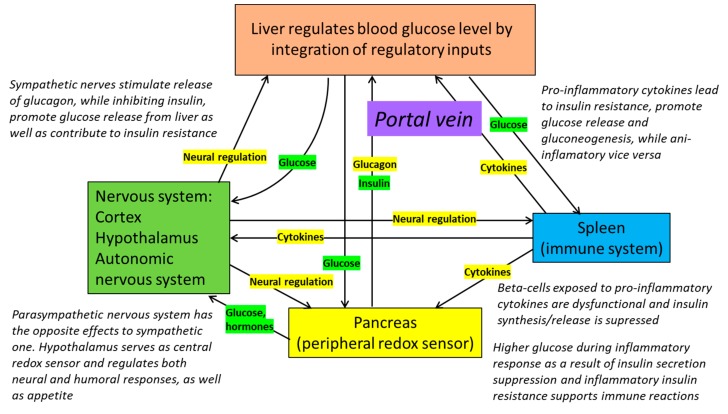
Role of redox sensors (pancreatic α and β cells), immune system and central nervous system in the regulation of blood glucose concentrations by liver. Glucose release or absorption by the liver integrates signals from nervous and immune systems, and peripheral redox sensors. The system is highly flexible and tunable, providing redox modulation that is dependent on actual needs. The other way around, glucose flows and redox state regulate the function of immune system [121]. Failure of feedback loops and distorted signaling—either from CNS (stress), the immune system (inflammation) or the malfunction of peripheral sensors—lead to excessive, uncontrolled (poorly controlled) glucose release and/or the activation of gluconeogenesis, leading to diabetes.

Glucose is a central metabolite and depending on its availability and metabolic need can play different roles in the organism. Glucose flows within the human body are strictly regulated and promptly adjustable in order to maintain metabolic flexibility and provide robust resistance to different types of stressing factors (Figure 4). The role of glucose is not limited to merely generating enough ATP (which is the case in conditions of glucose abundance and low stress), but more importantly, it is responsible for the emergency mechanisms of the maintenance of redox potential that is essential for survival in extreme situations (fight or flight reactions, infections, and poisoning).

Since TCA-cycle reactions and oxidative phosphorylation can be effectively maintained in almost absence of glucose by oxidation of fatty and amino acids, its residual amounts are—in catabolic conditions—redirected into the PPP to maintain redox balance in the cells. At the same time, the anabolic state requires redirecting excess glucose into energy production and biosynthesis (growth, proliferation, hypertrophy), when the primary life-supporting and life preserving needs ensured by glucose have already been met (“survival—first, growth—follows”). The proposed principle is helpful for understanding the regulation of glucose metabolism aimed primarily to maintain redox balance, especially in acute extreme conditions requiring prompt and massive antioxidant responses. Oxidative stress, infections/inflammation, starvation, exercise, aging and many other pathological conditions or processes decrease GSH/GSSG and NADPH/NADP^+^ ratios. Sensors sense these changes and drive glucose flows to compensate for these metabolic disturbances at the organismic level. In this regard insulin resistance develops as a protective “antioxidant” adaptive mechanism that stimulates glucose production and prevents its waste in order to cope with increased needs. When dysregulated and chronically over-activated, though, this leads to detrimental consequences caused by hyperglycemia [110].

The other important issue, which is often not taken into consideration by researchers, is that the presence of glucose stores in the form of glycogen or extracellular glucose availability provide enhanced resistance to oxidative stress. It is also possible that glucose—by strengthening the reductive power of NADPH-dependent antioxidant enzymes—prevents the activation of redox-sensitive regulatory factors such as Nrf2. That means that glucose nutritional overload affects physiological redox signaling, and the chronic over-activation of insulin signaling causes metabolic diseases, as described in detail [24]. On the other hand, severe glucose overload itself can serve as a source of oxidative stress in the cells through the participation of glucose in polyol pathway, over-the activation of NADPH oxidases and increased production of ROS by mitochondria. Exercise, caloric restriction, intermittent fasting, a ketogenic diet and some drugs have something in common—in that they deplete organisms’ glycogen stores [44], reduce glucose availability for the cells, restore the physiological redox signaling suppressed by chronic excessive glucose consumption and lead to a dramatic improvement of life- and health-spans in model organisms and humans (Figure 2). Metabolic changes caused by carbohydrate overload in the general population often take place far before clinically significant changes occur [43]. That is why relevant and sensitive instruments for the early detection of these metabolic shifts are needed.

## 12. Limitations of the Analysis and Directions of Further Research

In this review we presented mainly general principles of glucose metabolism in the cells and its importance for maintenance of redox balance. However, different cells in different tissues have their specific metabolic patterns, specific functions, and own physiological peculiarities. We tried to focus on the most important findings and inconsistencies in the literature from our point of view rather than focusing on biochemical details, different pathways, tissue differences and to show how the clinical and experimental evidence may be interpreted when the glucose will be considered as redox mediator rather than simply fuel “burned” to generate ATP. Glucose metabolism may differ substantially depending on the specifics of tissues and cells, proliferation activity, redox balance required to maintain the functions, expression and activities of involved enzymes. It is not possible to accurately describe all the evidence that is published in the literature, and we are aware that many important details may be missing from our analysis. Nevertheless, we hypothesize that convincing literature evidence indicates that glucose flows in the organism are primarily targeted to maintain redox homeostasis and counteract possible oxidative challenges.

It may be argued that the actual flow through PPP is relatively low in the brain (as has been shown by Gaitonde M. et al. [122]) and it only increases substantially to approximately 20% of total glucose utilization by neurons in cases of severe oxidative stress induced by hydrogen peroxide [123] or during experimental brain injury [124]. However, a closer look into the methods used in the studies reveals that non-physiologically high concentrations of glucose were used, namely 22.3 mM in the medium and 50.0 mM for perfusion in [123] and 23.9–26.9 mM plasma glucose after infusion in [124]). The flow of glucose into the PPP is inhibited in conditions of high NADPH/NADP^+^ ratio, therefore as soon as the levels of NADPH are restored, glucose is redirected into glycolysis or glycogen storage.

Our reconsiderations may provide better understanding of the physiology of glucose regulation in health and diseases and lead to the shift of the general paradigm of glucose-induced oxidative stress—which is, however true for hyperglycemia and diabetes—towards understanding the redox effects of glucose in a concentration-dependent manner. In other words, glucose maintenance at physiological levels is a fundamental mechanism of counteracting excessive oxidation due to its involvement in PPP and NADPH production. Endogenous antioxidant systems using glucose provide sufficient antioxidant defense in physiological conditions. Moreover, redox modulating agents that have some health benefits when supplemented often turn out to be rather prooxidant than antioxidant, and lead to the stimulation of endogenous antioxidant defenses and the improvement of glucose metabolism. Therapeutic approaches to applying antioxidant substances in order to reduce oxidative damage and the administration of compounds with the pure purpose to provide reducing equivalents appears weak [125] in contrast to often-high in vitro activities [126], and in comparison to existing endogenous antioxidant mechanisms based on glucose as a source of reducing power for the generation of NADPH and recycling oxidized glutathione and thiols, and can in some cases be deleterious since they may interfere with redox sensors—for example protein thiol groups, and cause dysregulations.

The other important issue is the lack of convenient, informative, sensitive and specific ways of glucose and/or glycogen determination in tissues for research and clinical use. As we suggested earlier, glycogen determination, preferably in a simple, affordable and noninvasive way could potentially be a good biomarker for redox biomedicine [44]. Unfortunately, there are too many technical issues preventing the development of such equipment for clinical use so far, but some efforts have been made to enable label-free glycogen estimation in *C. elegans*, an important animal model for metabolic and aging research [127].

## 13. Conclusions

Our critical analysis of the current literature unveiled significant controversies and an insufficient general understanding of metabolic role of glucose, particularly regarding redox homeostasis. The important function of glucose metabolism in the PPP for the maintenance of redox homeostasis is very often underestimated. In light of recent epidemiological evidence and advancements in the field of redox biology, it is hypothesized that a specific physiological function of glucose is its metabolism in the PPP, to provide stress resistance to unfavorable factors by the reduction in NADP^+^ to NADPH in order to maintain redox homeostasis and the correct functioning of immune cells. Meanwhile, glycolysis takes place in favorable redox conditions when glucose is in abundance. This approach and interpretation explains—in a simple way—the adverse metabolic effects and detrimental health consequences of nutritional carbohydrate overload, and provides new details in explaining the positive metabolic effects of intermittent fasting, caloric restriction, exercise, and a ketogenic diet. A better understanding of the evolutionary adaptations and biological role of glucose may serve as an important theoretical background for future experimental and clinical studies related to glucose metabolism, aging, and diabetes, as well as other adjacent fields.
